# Suboptimal Weight Loss 13 Years After Roux-en-Y Gastric Bypass Is Associated with Blunted Appetite Response

**DOI:** 10.1007/s11695-023-07028-w

**Published:** 2023-12-30

**Authors:** Siren Nymo, Julianne Lundanes, Kevin Eriksen, Marthe Aukan, Jens Frederik Rehfeld, Jens Juul Holst, Gjermund Johnsen, Hallvard Græslie, Bård Kulseng, Jorunn Sandvik, Catia Martins

**Affiliations:** 1 Obesity Research Group, Department of Clinical and Molecular Medicine, Faculty of Medicine and Health Sciences, Norwegian University of Science and Technology (NTNU), Forsyningssenteret, Prinsesse Kristinas gate 5, 7030 Trondheim, Norway; 2 Centre for Obesity and Innovation (ObeCe), Clinic of Surgery, St. Olav University Hospital, Trondheim, Norway; 3 Nord-Trøndelag Hospital Trust, Clinic of Surgery, Namsos Hospital, Namsos, Norway; 4 Department of Clinical Biochemistry, Rigshospitalet, University of Copenhagen, Copenhagen, Denmark; 5 NNF Center for Basic Metabolic Research and Department of Biomedical Sciences, The Panum Institute, University of Copenhagen, Copenhagen, Denmark; 6 Møre and Romsdal Hospital Trust, Clinic of Surgery, Ålesund Hospital, Ålesund, Norway; 7 Department of Nutrition Sciences, University of Alabama at Birmingham (UAB), Birmingham, AL USA

**Keywords:** Obesity, Hunger, Ghrelin, GLP-1, PYY

## Abstract

**Purpose:**

Bariatric surgery remains the most efficient treatment to achieve a sustained weight loss. However, a large proportion of patients experience suboptimal weight loss (SWL). The exact mechanisms involved remain to be fully elucidated, but the homeostatic appetite control system seems to be involved. The aim of this study was, therefore, to compare the plasma concentration of gastrointestinal hormones, and appetite ratings, between those experiencing SWL and optimal weight loss (OWL) after Roux-en-Y gastric bypass (RYGB).

**Materials and Methods:**

Fifty participants from the Bariatric Surgery Observation Study (BAROBS) experiencing either SWL or OWL (< or ≥ 50% of excess weight loss (EWL), respectively) > 13 years post-RYGB were compared to 25 non-surgical controls. Plasma concentrations of acylated ghrelin (AG), total glucagon-like peptide-1 (GLP-1), total peptide YY (PYY), cholecystokinin (CCK), and subjective ratings of hunger, fullness, desire to eat (DTE), and prospective food consumption (PFC) were assessed in the fasting and postprandial (area under the curve (AUC)) states.

**Results:**

Those experiencing OWL presented with higher basal AG and GLP-1 iAUC, and lower AG iAUC compared with SWL and controls. Additionally, both bariatric groups presented with higher PYY and CCK iAUC compared to controls. PFC tAUC was also lower in OWL compared to the SWL group. Total weight loss was positively correlated with GLP-1 tAUC and negatively correlated with fasting and tAUC DTE and PFC tAUC.

**Conclusions:**

SWL > 13 years post-RYGB is associated with lower basal ghrelin, as well as a weaker satiety response to a meal. Future studies should investigate the causality of these associations.

**Graphical Abstract:**

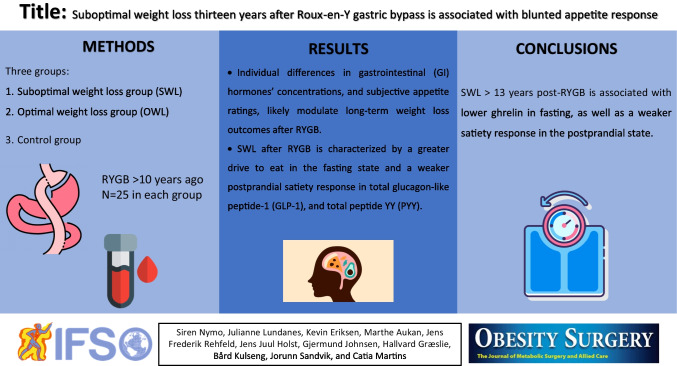

**Supplementary Information:**

The online version contains supplementary material available at 10.1007/s11695-023-07028-w.

## Introduction

Bariatric surgery is the most efficient treatment for inducing and maintaining clinically relevant weight loss (WL) and resolution of obesity associated medical problems in patients with severe obesity [1]. Roux-en-Y gastric bypass (RYGB), one of the most commonly performed bariatric procedures, can yield a total WL (TWL) of up to 38% of initial body weight 2 years post-operatively [2], or 57% excess WL (EWL) at 10 years follow-up [3]. However, weight regain (WR) over time is a concern [1], with up to 30% of patients experiencing WR and/or suboptimal weight loss (SWL) in the long run [4, 5]. Even though the mechanisms behind sustained WL post-RYGB are not fully understood, an exaggerated postprandial secretion of glucagon-like peptide-1 (GLP-1) and peptide YY (PYY), improved satiety, and reduced energy intake (EI) are likely to be involved [6–8].

The impact of RYGB on the plasma concentrations of ghrelin in the fasting state remains controversial [9], with some reporting a decrease [6, 10], and others an increase [8, 11]. A recent review concluded that ghrelin concentrations are usually reduced in the short term, but increased in the long term [12]. Similarly, a prospective study reported no change in hunger in the fasting state, 1 year post-RYGB [13], while others found increased hunger ratings [14, 15]. Regardless, ratings of prospective food consumption (PFC) in the postprandial state are reduced [15] and postprandial fullness increased post-RYGB [10, 13].

Despite the large number of studies describing changes in the plasma concentration of gastrointestinal (GI) hormones involved in appetite regulation post-RYGB, few have investigated inter-individual differences in relation to WL outcomes. Short-term report suppressed basal [11] and postprandial ghrelin [16], and increased postprandial GLP-1 [11, 16, 17] and PYY [11], to be associated with better WL maintenance. However, SWL 3 years post-RYGB was not associated with ghrelin plasma concentrations [18], and no differences in the concentrations of GI hormones were reported between those experiencing, or not, WR [19].

The primary aim of this study was, therefore, to compare the plasma concentration of GI hormones, and subjective appetite ratings, between those experiencing SWL and optimal weight loss (OWL), more than 10 years after RYGB, as well as a pre-operative control group. The secondary aim was to investigate the association between TWL, EWL, and WR, and the concentration of GI hormones, and appetite ratings in the surgical groups.

## Methods

### Study Design

This is a cross-sectional case-control study. Participants who underwent RYGB between 2003 and 2009 were invited to participate in this study and compared to a pre-operative control group, matched for pre-operative body mass index (BMI) of the surgical groups. An EWL of < or > 50% was used as criteria for SWL and OWL, respectively, and a WR of > 15% from nadir as criteria for significant WR [2, 20].

### Participants

Participants were recruited between 2019 and 2021, from the Bariatric Surgery Observation Study (BAROBS), an observational study in Central Norway. The participants post-bariatric surgery were divided into a SWL and an OWL group according to the definition previously provided. The control group comprised of participants on waiting list for bariatric surgery or enrolled in the DISGAP study (DIet versus Sleeve Gastrectomy and gastric bypass on APpetite) [10]. Exclusion criteria included pregnancy, breast feeding, medications, or medical conditions known to affect body weight, metabolism, or appetite, psychiatric diseases, eating disorders, and revisional surgery.

Both the BAROBS and DISGAP studies were approved by the regional ethics committee (REK 2017/1828-21 and 2019/252, respectively). Additionally, the DISGAP study was registered in clinical trials (NCT04051190). All participants provided written informed consent in line with the Helsinki Declaration, before entering the studies.

### Bariatric Procedure

The RYGB procedure was performed laparoscopically according to the Lönroth technique, with a pouch of 15–30 mL, biliopancreatic limb of 40–60 cm, and an antecolic, antegastric alimentary limb of 100 cm or 150 cm, depending on BMI < or > 50 kg/m^2^. A linear stapler was used for the anastomosis, and the mesenteric defects were not closed [21].

## Assessments

Anthropometric measurements were taken in the fasting state (12 h), and appetite markers were assessed before and after a standardized liquid breakfast.

### Body Weight and Composition

Body weight and composition were assessed with air-displacement plethysmography (BodPod, Cosmed, Concord, CA, USA), using the Brozeq equation [22].

Ideal weight was the weight corresponding to a BMI of 25 kg/m^2^, and weight at nadir was the lowest weight registered at the hospital the first 2 years post-surgery. %TWL, %EWL, and %WR were estimated using standard equations [23].

### Appetite Markers

Blood samples and appetite ratings were collected in fasting, and at different timepoints for 2.5 h after a standardized liquid breakfast (200 mL of Diben shake (Fresenius Kabi, Bad Homburg, Germany), nutritional composition per 200 mL: 300 kcal, 15 g protein, 14 g fat, and 26 g carbohydrates). Plasma samples were analyzed for active ghrelin (AG) and total PYY using a Human Metabolic Hormone Magnetic Bead Panel (LINCOplex Kit, Millipore, St Louis, MO, USA), as well as total GLP-1 and CCK using “in-house” RIA methods [24, 25]. All the samples from the same participant were analyzed in the same plate. Subjective feelings of hunger, fullness, desire to eat (DTE), and PFC were measured using a validated visual analogue scale [26].

## Power Calculations

This study was originally powered to look at differences in hedonic hunger, as measured by the power of food scale (PFS), between groups. Using data from Schultes et al. [27], and assuming that controls (pre-operative) and those with SWL had the same PFS score (2.8) and those with OWL a lower PFS (2.2), for a SD of 0.7, a power of 80%, and a significance level of 5%, 21 participants/group would be needed. Assuming a drop-out rate of 20%, 25 participants/group were deemed necessary (75 in total).

For the present study, the main outcome variable was 3 h postprandial GLP-1 response. Based on the study by le Roux et al. [28], we estimated the AUC for GLP-1 to be 2000 pmol/L*min in the pre-operative controls, 3500 pmol/L*min in the SWL group, and 8500 pmol/L*min for the OWL group. For a SD of 567 pmol/L*min, at a power of 90% and a significance level of 5%, the estimated sample size was 6 subjects/group.

## Statistical Analysis

Statistical analysis was performed using IBM SPSS Statistics 27 (SPSS In., Chicago, IL, USA), and data presented as mean ± SD for arthrometric variables and estimated marginal mean ± SEM for the other variables. All variables were checked for normality with the Shapiro-Wilk test and visual inspection of histograms and Q-Q plots. Statistical significance was assumed at *P* < 0.05, unless otherwise stated. Differences between groups were tested with a linear mixed model, with fixed effects for group. Adjusting for age and pre-operative BMI did not change the results; therefore, unadjusted results are presented.

We were unable to collect blood samples from two participants in the control group and these were excluded from the analysis of GI hormones. The trapezoidal rule was applied to calculate total area under the curve (tAUC) from 0 to 150 min. Correlation between appetite markers, and %EWL, %TWL, and %WR was performed with Pearson’s or Spearman’s correlation, depending on the normality of the data. In addition, linear regression was used to determine if appetite variables were significant predictors of long-term WL outcomes (TWL and EWL) after adjusting for age, sex, and pre-operative BMI.

## Results

### Participants

A total of 50 participants from the BAROBS study enrolled in this study, as well as 25 controls from the DISGAP study. The bariatric group comprised of 25 participants with SWL and 25 with OWL. Participants had an average age of 49.0 ± 9.9 years and a pre-operative BMI of 43.2 ± 4.6 kg/m^2^. The control group was significantly younger than the bariatric groups. The SWL group presented with higher fat mass (FM)% compared to the OWL group, as well as lower %EWL and %TWL and higher %WR compared to the OWL group (*P* < 0.001, for all) (see Table 1).

**Table 1:** General characteristics of the participants

	All participants (*n* = 75)	SWL (*n* = 25)	OWL (*n* = 25)	Controls (*n* = 25)
Female, *n* (%)	*59* (79)	*20* (80)	*23* (92)	*16* (64)
Weight, kg	108.0 ± 28.3	124.9 ± 19.2^b^	75.6 ± 13.3^b,a^	126.6 ± 15.3^a^
BMI, kg/m^2^	37.3 ± 8.4	43.1 ± 5.7^a^	27.0 ± 3.9	42.0 ± 3.2^b^
Pre-operative BMI, kg/m^2^	43.2 ± 4.6	46.3 ± 5.4^a,^	41.4 ± 3.5^a^	
Age, years	49.0 ± 9.9	50.5 ± 6.3^a^	52.5 ± 9.5^b^	43.7 ± 11.9^a,b^
FM, %	45.0 ± 8.9	51.3 ± 4.4^a^	35.7 ± 6.4^a,b^	48.0 ± 5.2^b^
%EWL	52.0 ± 41.0	31.9 ± 32.8^a^	70.1 ± 40.0^a^	
%TWL	21.5 ± 16.7	13.4 ± 13.6^a^	28.4 ± 15.6^a^	
%WR	16.7 ± 20.2	24.1 ± 20.8^a^	10.0 ± 16.4^a^	
Weight increase from nadir, kg	16.5 ± 20.0	26.9 ± 15.0^a^	6.1 ± 15.0^a^	

Data presented as mean ± SD. Means with the same subscript letter are significantly different. ^a,b^*P* < 0.001. *BMI*, body mass index; *FM*, fat mass; *EWL*, excess weight loss; *TWL*, total weight loss; *SWL*, suboptimal weight loss; *OWL*, optimal weight loss; *WR*, weight regain

### Plasma Concentrations of Gastrointestinal Hormones

**Fig. 1:**
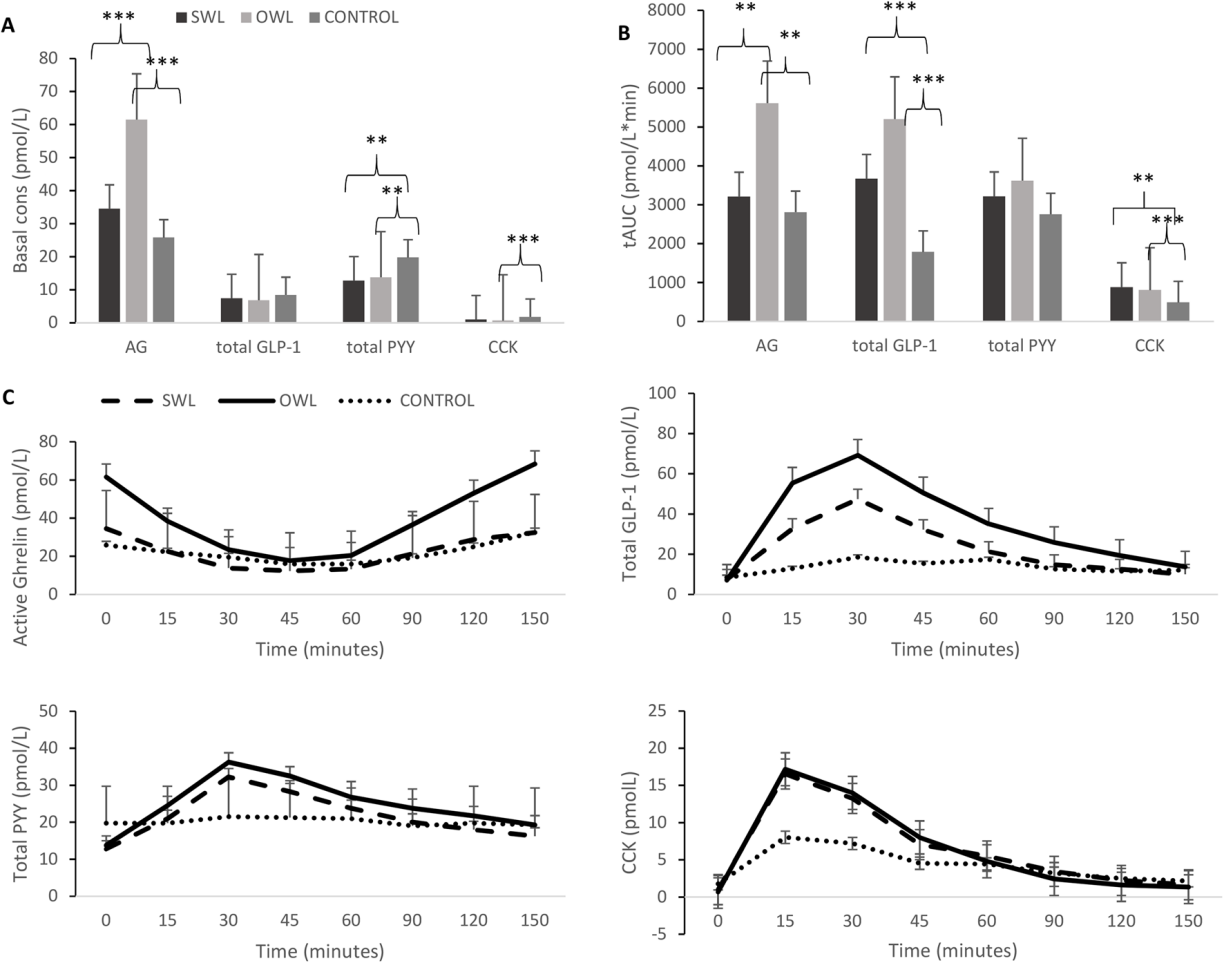
Basal (**A**) and postprandial (**B**) plasma concentrations of gastrointestinal hormones and profile over time (**C**) for suboptimal weight loss, optimal weight loss, and control groups. Data presented as estimated marginal means ± SEM. SWL, suboptimal weight loss. OWL, optimal weight loss. AG, active ghrelin; GLP-1, glucagon-like peptide-1; PYY, peptide YY; CCK, cholecystokinin; tAUC, total area under the curve. ***P < 0.001, **P < 0.01, and *P < 0.05 denote significant differences between groups

Basal and postprandial concentrations of AG were higher in the OWL compared with the SWL and controls (basal *P* < 0.001; postprandial *P* = 0.001). Postprandial (tAUC) GLP-1 was higher in bariatric groups versus controls (*P* < 0.001 for OWL and *P* < 0.01 for SWL). Basal plasma concentration of PYY was lower (*P* = 0.001 and *P* < 0.01, respectively), in OWL and SWL versus controls. Basal plasma concentrations of CCK were lower in OWL versus controls (*P* < 0.01), while both tAUC higher in the OWL and SWL groups versus controls (tAUC *P* < 0.01 and *P* = 0.001, respectively) (see Table 2 and Fig. 1).

**Table 2:** Mean basal and postprandial concentrations of gastrointestinal hormones across groups

	SWL	OWL	Controls	*P* value
Basal AG (pmol/L)	33.9 ± 23.86^b^	61.6 ± 24.7^a,b^	25.7 ± 17.0^a^	< 0.001
AG tAUC (pmol/L*min)	3208.3 ± 2229.0^d^	5844.0 ± 2573.0^c,d^	3191.9 ± 2344.0^c^	< 0.001
Basal GLP-1 (pmol/L)	7.4 ± 5.7	6.8 ± 5.6	8.4 ± 4.9	0.325
GLP-1 tAUC (pmol/L*min)	3667.2 ± 2015.7^e^	5201.9 ± 2847.7^a^	1786.2 ± 1296.9^e,a^	< 0.001
Basal PYY (pmol/L)	12.7 ± 4.9^c^	13.3 ± 5.3^e^	17.6 ± 10.5^c,e^	0.026
PYY tAUC (pmol/L*min)	3213.9 ± 1325.3	3620.6 ± 1682.2	2754.7 ± 2754.7	0.135
Basal CCK (pmol/L)	1.0 ± 0.7	0.7 ± 0.6^e^	1.8 ± 1.8^e^	0.006
CCK tAUC (pmol/L*min)	877.6 ± 452.5^c^	807.6 ± 342.9^e^	490.1 ± 390.7^c,e^	0.002

Data presented as estimated marginal means ± SD. Conversion from metric to SI units has been applied as follows: ghrelin pg/mL × 0.3 = pmol/L, PYY pg/mL × 0.25 = pmol/L

*SWL*, suboptimal weight loss; *OWL*, optimal weight loss; *AG*, acylated ghrelin; *CCK*, cholecystokinin; *GLP-1*, total glucagon-like peptide-1; *PYY*, total peptide YY; *tAUC*, total area under the curve. *P* value for main effect of group. Mean values with equal superscript letters denote significant differences between groups: ^a,b^*P* < 0.001; ^c,d^*P* = 0.001; and ^e,f^*P* < 0.01

### Appetite Ratings

**Fig. 2:**
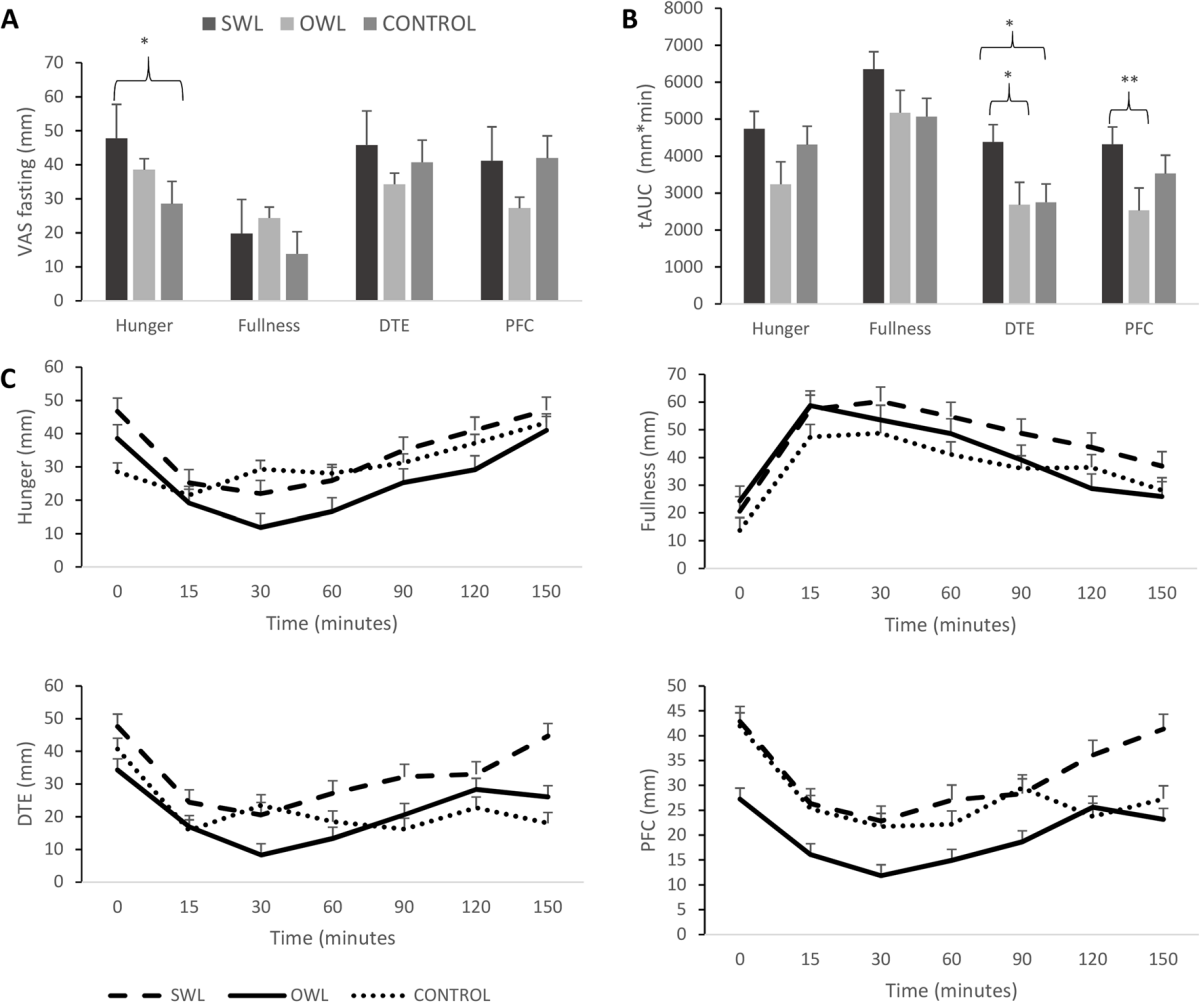
Subjective appetite ratings in the fasting (**A**), and postprandial states (**B**) and profiles over time (**C**) for suboptimal weight loss, optimal weight loss, and control groups. Data presented as means ± SEM. SWL, suboptimal weight loss. OWL, optimal weight loss; DTE, desire to eat; PFC, prospective food consumption; tAUC, total area under the curve. *P < 0.05 and **P < 0.01 denote significant differences between groups

There was a tendency for hunger ratings in the fasting state to be higher in the SWL group compared to controls (*P* < 0.057), and in the SWL compared with the OWL group (*P* < 0.05). DTE in the postprandial state was higher in the SWL versus both OWL and control groups (tAUC *P* < 0.05 for both), while postprandial PFC was lower in the OWL versus SWL group (tAUC *P* < 0.01) (see Table 3 and Fig. 2).

**Table 3:** Subjective appetite ratings, in fasting and postprandial states, across group

Controls	SWL		OWL	*P* value
Fasting hunger (mm)	47.8 ± 24.4^a^	38.5 ± 27.6	28.6 ± 30.9^a^	0.057
tAUC hunger (mm*min)	4735.0 ± 2717.2	3238.4 ± 2388.7	4308.5 ± 4308.5	0.157
Fasting fullness (mm)	19.8 ± 21.5	24.3 ± 20.0	13.8 ± 16.7	0.234
tAUC fullness (mm*min)	6346.6 ± 2815.9	5173.4 ± 3290.2	5066.4 ± 2605.0	0.318
Fasting DTE (mm)	45.8 ± 27.6	34.3 ± 30.4	40.7 ± 30.0	0.392
tAUC DTE (mm*min)	4378.1 ± 2710.4^a,b^	2684.8 ± 2689.1^b^	2748.7 ± 2481.1^a^	0.041
Fasting PFC (mm)	41.2 ± 20.4	27.3 ± 17.4	42.0 ± 36.2	0.094
tAUC PFC (mm*min)	4315.2 ± 2547.3^d^	2530.0 ± 1895.2^d^	3526.4 ± 2457.1	0.029

Data presented as estimated marginal means ± SD. *SWL*, suboptimal weight loss; *OWL*, optimal weight loss; *DTE*, desire to eat; *PFC*, prospective food consumption; *tAUC*, total area under the curve. *P* value for main effect of group. Mean values with equal superscript letters denote significant differences between groups. ^a,b,c^*P* < 0.5. ^d^*P* < 0.01

### Correlations

**Fig. 3:**
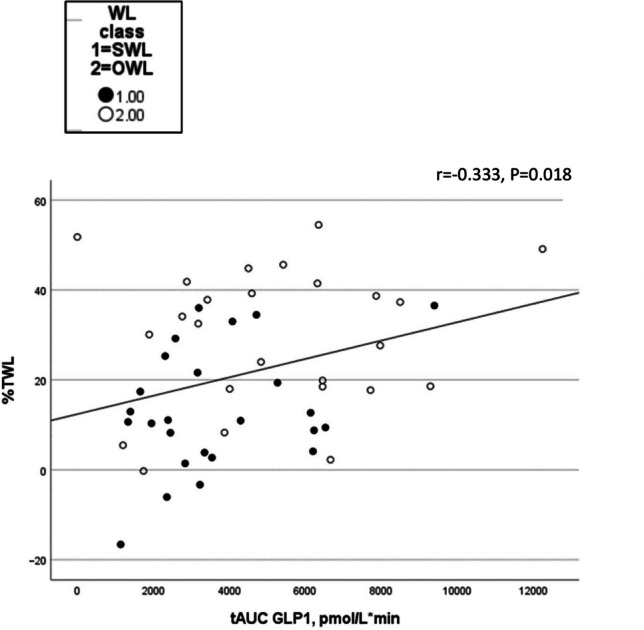
Scatterplots for the association between postprandial GLP-1 and total weight loss in the bariatric surgery groups. tAUC, total area under the curve; TWL, total weight loss; GLP-1, glucagon-like peptide-1; WL, weight loss; SWL, suboptimal weight loss; OWL, optimal weight loss

**Fig. 4:**
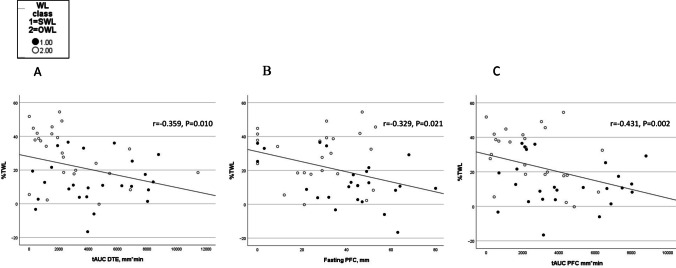
Scatterplots for the association between postprandial DTE and PFC and total weight loss in the bariatric surgery groups. DTE tAUC and TWL (**A**); PFC in the fasting state and TWL (**B**); and PFC tAUC and TWL (**C**). tAUC, total area under the curve; TWL, total weight loss; WL, weight loss; SWL, suboptimal weight loss; OWL, optimal weight loss; PFC, prospective food consumption; DTE, desire to eat

There was a positive association between GLP-1 tAUC, as well as basal PYY, and TWL and EWL, and a negative association between GLP-1 and PYY tAUC and WR. A negative correlation between DTE tAUC, as well as fasting and tAUC PFC, and TWL and EWL was also seen (see Supplementary table 1 and Figs. 3 and 4, only correlations between appetite variables and TWL are presented).

### Regression Analyses

Postprandial concentrations of GLP-1 and PYY were not significant predictors of TWL or EWL after adjusting for age, sex, and pre-operative BMI. However, ratings of PFC in the fasting and postprandial states, as well as DTE in the postprandial state, were significant predictors of both TWL and EWL, even after adjusting for confounders, with the regression models explaining between 15 and 33% of the variation in long-term WL outcomes (Supplementary table 2 and 3).

## Discussion

This study aimed to compare GI hormones’ concentrations and appetite ratings between patients with SWL and OWL more than 13 years after RYGB, as well as a non-surgical control group. Patients with SWL had lower concentrations of AG, and a lower postprandial GLP-1 response, compared with both the OWL and control groups, and higher postprandial ratings of PFC compared to OWL. Additionally, both bariatric groups presented with higher PYY and CCK concentrations in the postprandial period compared with the controls. In line with this, TWL and EWL increased with increasing postprandial GLP-1 and PYY concentrations, and with decreasing ratings of PFC and DTE.

### Gastrointestinal Hormones

The lower AG concentrations seen in the SWL group are likely a result of the higher body weight seen in this group, as lower ghrelin plasma concentrations have been reported in individuals with obesity compared with those with a normal weight [29], and WL is accompanied by increased ghrelin concentrations [30–32]. This also supports our findings of a positive association between AG and long-term WL outcomes. The inconsistent findings previously reported on the impact of RYGB on AG plasma concentration [8, 9, 11, 33] are likely to reflect differences in follow-up time and hormonal fraction measured, as well as surgical procedure [9], particularly the residual amount of intact fundus [34], where most of ghrelin is produced [34].

GLP-1 response to the meal was lower in the SWL group, indicating that these individuals are likely experiencing a lower satiety compared with the OWL group. Additionally, a positive association was seen between GLP-1 postprandial response and long-term WL outcomes. An increase in GLP-1 in the postprandial state has systematically been described after RYGB [7, 8, 35], and suggested as one of the mechanisms responsible for sustained WL following this bariatric procedure.

PYY plasma concentrations were higher in the bariatric groups compared to the controls, but no differences were seen between the OWL and SWL groups. This is in line with previous studies reporting an increase in PYY concentrations after RYGB [7, 8]. However, and in line with previous literature [8], PYY concentrations were not correlated with WL outcomes. The increased GLP-1 and PYY concentration post-RYGB likely results from the anatomical changes following this procedure, with more undigested food reaching the ileum faster [35].

Even though inter-individual variations in GLP-1 secretion have been reported after RYGB [36, 37], from our knowledge, no previous study had reported an association between this satiety hormone and long-term WL outcomes. Results from the present study indicate that a lower postprandial GLP-1 response might be associated with SWL. However, because postprandial GLP-1 response decreases with increasing BMI and FM [29], it remains to be ascertained if the lower postprandial GLP-1 concentrations seen in the SWL group are a cause or consequence of their higher body weight. More research is clearly needed to ascertain the direction of causality.

### Appetite Ratings

In the present study, the SWL group presented with higher hunger ratings in the fasting state compared with controls, higher postprandial PFC compared with the OWL group, and higher DTE compared to both OWL and controls. Additionally, a negative association was seen between DTE and PFC ratings, and WL outcomes. Even though a reduction in hunger ratings [13], and DTE [33], has previously been reported following RYGB, hunger ratings were reported not to be associated with WL outcomes post-RYGB in another study [14]. The differences in hunger ratings between groups could be explained by the differences in eating behaviour and dietary intake as found in our previously published studies [38, 39], showing an association between increased preference and reward for high-fat food and increased hedonic hunger, in the same sample reported here. Ratings of postprandial fullness have been described to increase following RYGB and believed to facilitate WL [13, 14, 40]. In line with this, a trend towards lower postprandial fullness ratings was seen in the SWL compared with the OWL group in the present study. Differences in the energy and macronutrient composition of the test meal, post-operative follow-up time, and method to assess subjective appetite are likely to have contributed to some of the differences observed.

The hedonic appetite control system is also likely involved in modulating long-term WL outcomes following RYGB. Hedonic hunger has been found to predict WL 2 years after bariatric surgery [41], and we have recently reported that SWL 13 years after RYGB was associated with increased preference and reward for high-fat food and increased hedonic hunger, in the same sample reported here [38]. This study presents with several strengths. First, it reports long-term results with a follow-up time of at least 13 years post-RYGB. Second, both subjective appetite ratings and the plasma concentration of GI hormones were measured, in the fasting and postprandial states. Last, the study had a non-surgical control group. However, this study also has some limitations. The main limitation is its cross-sectional design, which does not allow for conclusions to be drawn regarding direction of causality between blunted satiety and SWL post-RYGB. Another limitation is the use of a multikit to analyze ghrelin and PYY, which is less accurate than optimized assays for each hormone. Finally, even though the study has enough power to detect differences in postprandial GLP-1 plasma concentrations between groups, it might not have enough power to identify differences in other appetite-related variables among groups.

The results of the present analysis are of clinical relevance, as individual differences in the concentration of GI hormones and appetite ratings are likely to be involved in modulating long-term WL outcomes post-RYGB. SWL is characterized by a greater drive to eat (PFC and DTE) and a weaker postprandial satiety response (GLP-1 and PYY). Subjective appetite ratings are relatively easy and cheap to measure and could potentially be used as a screening tool to identify those at risk of SWL that could be then offered a more intensive follow-up, or even GLP-1 analogues [42].

In conclusion, SWL 13 years after RYGB is associated with lower ghrelin plasma concentrations, and a weaker postprandial GLP-1 response, as well as a greater drive and motivation to eat. Future studies should have a longitudinal design to clarify the cause-effect relationship of the previously described associations.

### Supplementary Information

Below is the link to the electronic supplementary material.

Supplementary file1 (DOCX 22 KB)

## Abbreviations

BAROBS: Bariatric Surgery Observation Study
BMI: Body mass index
CCK: Cholecystokinin
DISGAP: DIet versus Sleeve Gastrectomy and gastric bypass on Appetite
DTE: Desire to eat
EI: Energy intake
EWL: Excess weight loss
GI: Gastrointestinal
GLP-1: Glucagon-like peptide-1
OWL: Optimal weight loss
PFC: Prospective food consumption
PYY: Peptide YY
REK: Regional ethics committee
RYGB: Roux-en-Y gastric bypass
SWL: Suboptimal weight loss
tAUC: Total area under the curve
TWL: Total weight loss
WL: Weight loss
WR: Weight regain
