# Supplementary material for: Suboptimal Weight Loss 13 Years After Roux-en-Y Gastric Bypass Is Associated with Blunted Appetite Response
Source: Obes Surg. 2023 Dec 30;34(2):592–601. doi: 10.1007/s11695-023-07028-w (PMC10811108; doi:10.1007/s11695-023-07028-w)
Supplement: Supplementary file 1 — Supplementary file1 (DOCX 22 KB) [file 11695_2023_7028_MOESM1_ESM.docx]

| Supplementary table 1. Correlation between long-term weight loss outcomes and appetite markers in the surgical groups | | | | |
| --- | --- | --- | --- | --- |
|  | TWL | | |  |
|  | r | | P |  |
| Basal AG, pmol/L | | 0.079 | 0.880 |  |
| AG tAUC, pmol/L*min | | -0.052 | 0.722 |  |
| Basal GLP-1, pmol/L | | 0.062 | 0.671 |  |
| GLP-1 tAUC, pmol/L*min | | ***0.333*** | ***0.018*** |  |
| Basal PYY, pmol/L | | ***0.317*** | ***0.026*** |  |
| PYY tAUC, pmol/L*min | | 0.211 | 0.140 |  |
| Basal CCK, pmol/L | | -0.177 | 0.224 |  |
| CCK tAUC, pmol/L*min | | -0.002 | 0.991 |  |
| Fasting hunger, mm | | 0.135 | 0.355 |  |
| Hunger tAUC, mm*min | | -0.091 | 0.531 |  |
| Fasting fullness, mm | | 0.177 | 0.223 |  |
| Fullness tAUC, mm*min | | 0.031 | 0.832 |  |
| Fasting DTE, mm | | 0.022 | 0.879 |  |
| DTE tAUC, mm*min | | ***-0.359*** | ***0.010*** |  |
| Fasting PFC, mm | | ***-0.329*** | ***0.021*** |  |
| PFC tAUC, mm*min | | ***-0.431*** | ***0.002*** |  |
| AG: acylated ghrelin. CCK: cholecystokinin. DTE: desire to eat. GLP-1: glucagon-like peptide 1. P: p-value.  PFC: prospective food consumption. PYY: peptide YY. tAUC: total area under the curve. WR: weight regain. | | | | |

| Supplementary table 2. Multivariate linear regression models predicting weight loss outcomes 13 years after Roux-en-Y gastric from GI hormone plasma concentrations | | | |
| --- | --- | --- | --- |
| Models | ß-coefficient (95% CI) | P value | Adjusted R^2^ |
| 1. TWL%   Multivariate model  Constant  Age  Sex  Preoperative BMI  GLP-1 Fasting   1. EWL%   Multivariate model  Constant  Age  Sex  Preoperativ BMI  GLP-1 AUC   1. TWL%   Multivariate model  Constant  Age  Sex  Preoperativ BMI  PYY AUC   1. EWL%   Multivariate model  Constant  Age  Sex  Preoperativ BMI  PYY AUC | 21.906 (-33.720, 77.531)  0.401 (-0.282, 1.084)  -5.763 (-18.999,7.472)  -0.479 (-1.401, 0.444)  0.001 (-0.001, 0.003)  103.132 (-28.344, 234.608)  0.903 (-0.711, 2.517)  -11.628 (-42.912, 19.656)  -2.266 (-4.44, -0.085)  0.003 (-0.002,0.008)  19.900 (-39.595, 76.394)  0.582 (-0.069, 1.234)  -5.643 (-19.208, 7.922)  -0.538 (-1.475, 0.399)  1.1E^±5^(-0.001,0.001)  98.396 (-35.202, 231.995)    1.345 (-0.196, 2.885)  -11.375 (-43.452, 20.703)  -2.412 (-4.628, -0.197)  -1.0E^±5^ (-0.002, 0.002) | 0.069  0.432  0.243  0.385  0.302  0.299  0.164  0.121  0.266  0.458  0.042  0.289  0.108  0.482  0.079  0.407  0.253  0.981  0.027  0.145  0.086  0.476  0.034  0.992 | 0.099  0.016  0.077  0.143 |
| TWL, Total weight loss; EWL, excess weight loss; tAUC, total areal under the curve; GLP-1, glucagon like peptide-1; PYY, polypeptide YY. Variance inflation factors (VIF) <1.1. | | | |

| Supplementary table 3. Multivariate linear regression models for predicting weight loss outcomes 13 years after Roux-en-Y gastric bypass from subjective appetite feelings | | | |
| --- | --- | --- | --- |
| Models | ß-coefficient (95% CI) | P value | Adjusted R^2^ |
| 1. TWL%   Multivariate model  Constant  Age  Sex  Preoperative BMI  PFC fasting   1. EWL%   Multivariate model  Constant  Age  Sex  Preoperative BMI  PFC fasting   1. TWL%   Multivariate model  Constant  Age  Sex  Preoperative BMI  PFC tAUC   1. EWL%   Multivariate model  Constant  Age  Sex  Preoperative BMI  PFC tAUC   1. TWL%   Multivariate model  Constant  Age  Sex  Preoperative BMI  DTE tAUC   1. EWL%   Multivariate model  Constant  Age  Sex  Preoperative BMI  DTE tAUC | 24.275 (-25.025, 73.576)  0.670 (0.148, 1.192)  -5.069 (-16.837, 6.698)  -0.561 (-1.384, 0.261)  -0.3 (-0.468, 0.057)  109.21 (-7.943, 226.586)  1.513 (0.271, 2.754)  -9.523 (-37.514, 18.467)  -2.4 (-4.3, -0.4)  -0.701(-1.190, -0.212)  48.592 (-7.845, 105.030)  0.357 (-0.219, 0.932)  -8.792 (-21.453, 3.868)  -0.667 (-1.532, 0.199)  -0.003 (-0.004, -0.001)  170.332 (38.233, 302.431)  0.774 (-0.573, 2.121)  -19.243 (-48.876, 10.391)  -2.732 (-4.758, -0.706)  -0.06 (-0.011, 0.020)  47.169 (-10.631, 104.969)  0.404 (-0.175, 0982)  -7.919 (-20.694, 4.982)  -0.748 (-1.639, 0.144)  -0.002 (-0.004, 0)  166.609 (30.973, 302.245)  0.892 (-0.466, 2.250)  -17.036 (-47.015, 12.943)  -2.934 (-5.025, -0.843)  -0.005 (-0.009, 0.001) | 0.002  0.326  0.013  0.390  0.176  0.014  <0.001  0.067  0.018  0.497  0.006  0.006  0.006  0.090  0.218  0.169  0.128  0.010  <0.001  0.013  0.253  0.198  0.009  0.006  0.011  0.107  0.167  0.218  0.098  0.022  0.002  0.017  0.192  0.258  0.007  0.015 | 0.256  0.326  0.204  0.276  0.179  0.249 |
| TWL, Total weight loss; EWL, excess weight loss; tAUC, total areal under the curve; PFC, prospective food consumption; DTE, desire to eat. Variance inflation factors (VIF) <1.1. | | | |
